# Distribution of the *Najas* species in Lithuania (Hydrocharitaceae): Revised data from the 19^th^ century onwards

**DOI:** 10.3897/BDJ.14.e174747

**Published:** 2026-01-06

**Authors:** Liucija Kamaitytė-Bukelskienė, Zofija Sinkevičienė

**Affiliations:** 1 State Scientific Research Institute Nature Research Centre, Vilnius, Lithuania State Scientific Research Institute Nature Research Centre Vilnius Lithuania

**Keywords:** distribution, herbarium specimens, Hydrocharitaceae, *
Najas
*, submerged macrophytes

## Abstract

The article presents data on the distribution of four *Najas* species in Lithuania, starting from the first records at the beginning of the 19^th^ century. The work is based on a review of specimens stored in Lithuanian herbaria, paying special attention to the localities indicated on the labels or mentioned in publications. *Najas marina* and *Najas major* were first presented in Lithuania as separate species, occurring in different waterbodies. The wide distribution of these species and in several locations observed abundance, indicating that they do not require protection. However, monitoring of future distribution changes would be recommended due to possible invasiveness. The first known locality of *Najas minor* was corrected, based on information in herbarium labels and literature sources. Based on the acquired data, strict protection is still required for the rare species, *Najas minor* and particularly *Najas flexilis*, as the known localities in Lithuania are the southernmost extent of its distribution range. With minor exceptions, the distribution of all recorded *Najas* species within Lithuania is restricted to waterbodies in the Baltic Uplands, extending from the south to the northeast of the country.

## Introduction

The genus *Najas* has historically been placed in the monotypic family Najadaceae, based on its underwater pollination system (Takhtajan 1966, Cronquist 1981, Tomlinson 1982). With the help of molecular studies (Tanaka et al. 1997, Les et al. 1997, Chen et al. 2012), it was assigned to the family Hydrocharitaceae (POWO 2025). The genus was split into subgenus Najas, representing dioecious, robust and spiny plants and subgenus Caulinia, consisting of mostly monoecious, delicate and insignificantly armed species (Tzvelev 1979, Triest 1988). The database POWO (2025) includes 39 accepted species of the genus *Najas*, with five native to Europe: *N. tenuissima* (A. Braun ex Magnus) Magnus, *N. minor* All., *N. flexilis* (Willd.) Rostk. & W.L.E. Schmidt, *N. major* All. and *N. marina* L. Four species are known to be introduced: *N. graminea* Delile, *N. gracillima* (A.Braun ex Engelm.) Magnus, *N. guadalupensis* (Spreng.) Magnus and *N. chinensis* N.Z.Wang (Hussner 2012, Hrivnák et al. 2019).

*Najas marina* L., representing the genus and the subgenus Najas, is a robust, annual, submerged plant with an almost cosmopolitan distribution (Hulten and Fries 1986, Ito et al. 2017). It has usually been considered as one highly variable species *N. marina* sensu lato (hereinafter called s.l.), composed of taxa of different ranks: species (Allioni 1773, Gorski 1830, Lindberg 1932), subspecies (Viinikka 1976, Triest 1988) or varieties and forms (Braun 1864, Rendle 1899, Triest 1988). For the European part of the range, Viinikka (1976) recognised two taxa based on different karyotypes, corresponding to N. marina subsp. major (cytodeme A) and N. marina subsp. marina (cytodeme B). Karyological differentiation was supported by different morphological characters, different distribution and habitat preferences. The major was characterised by wider leaves and larger fruits and preferred fresh, warm and nutrient-rich waters in central Europe. Distribution of the second subsp. marina was associated with more northerly regions around the Baltic Sea, with few localities in the Alps, occurring in both fresh and brackish waters. In the same work, Viinikka (1976) stated that the type specimens of *N. marina* L. and with *N. intermedia* (Wolfg.) Gorski belong to the same taxon and synonymised N. marina subsp. marina with *N. intermedia*. However, Casper (1979) and Triest (1988) related N. marina subsp. marina with *N. major* All. Bräuchler (2015), in the last revision of *N. marina* s.l., attributed N. marina subsp. marina to separate species *N. major* All. (karyotype A, sensu Viinikka (1976)) and N. marina subsp. intermedia – to *N. marina* L. (karyotype B, sensu Viinikka (1976)). All names and synonyms of both subspecies and subordinate taxa attributed by Triest (1988) belong to the corresponding species by Bräuchler (2015). Recent molecular studies by Rüegg et al. (2017) have identified two genetically distinct lineages in *N. marina* s.l. that correspond to the respective karyotypes of Viinikka (1976). Similarly, phylogenetic studies of *Najas* by Ito et al. (2017) revealed two distinct clades for *N. marina* s.l. However, due to the overlap of morphological features, the presence of two species is still under discussion (Ito et al. 2017).

Back in the 19^th^ century, *Najas major* and the the new described species *N. intermedia* were recorded in Lithuania (Wolfgang 1824, Gorski 1830). However, later Lithuanian botanical issues (Snarskis 1954, Šarkinienė 1963a) treated *N. major* as a synonym of *N. marina*. Although this species was divided into two varieties, a typical *marina* and var. intermedia, the differences between these two taxa were overlooked, probably due to their rarity at the time. *N. marina* s.l. was Red-listed for quite a long time (Balevičius 1992, Rašomavičius 2007). In the Flora of the Baltic countries (Kuusk et al. 2003), only the occurrence of N. marina subsp. intermedia was reported for Lithuania, Latvia and Estonia, although Tzvelev (1979) indicated that two separate taxa (*N. marina* and *N. major*) were characteristic for this region. The concept of two separate species was followed in the latest Latvian account of *Najas* species (Suško et al. 2022).

*Najas minor* All., representing the subgenus Caulinia, is native to Europe, western Asia and northern Africa (Triest 1988) and is introduced in North America (Les et al. 2015, POWO 2025). Although this species is classified globally and in Europe as Least Concern (LC) by IUCN Criteria, a tendency towards decline in habitat area, quality and number of mature individuals has been observed (Lansdown 2014). The species is rare and listed as endangered in Lithuania and Latvia, but has not been recorded in Estonia (Kuusk et al. 2003, Rašomavičius 2021, Suško et al. 2022).

*Najas flexilis* (Willd.) Rostk. & W.L.E. Schmidt is a species with circumpolar, boreal-montane distribution, but is much more frequent in North America than in Europe and Asia (Hulten and Fries 1986, Preston and Hill 1997). Due to its significant decline in Europe, including extinction in Germany, Switzerland and Poland, *N. flexilis* was assessed as vulnerable (VU) according to IUCN Criteria (Landsdown 2011) and listed in Annexes II and IV of the European Council or "Habitats" Directive (Council Directive 92/43/EEC). Within the European range, the species is most frequent and studied in Scotland and Ireland (Wingfield et al. 2004, Wingfield et al. 2005, Wingfield et al. 2006, Gunn and Carvalho 2020, Roden et al. 2021). Les et al. (2015) recognised that *N. flexilis* s.l. consists of two genetically distinct cryptic species: the diploid species *N. flexilis*, which has thicker seeds and the tetraploid species *N. canadensis* Michx., which has thinner seeds. The study by Les et al. (2015) also identified *N. canadensis* in Latvia. This neighbouring country is characterised as having the second largest number of localities of *N. flexilis* s.l. in Europe (Suško et al. 2022), although this taxon is Red-listed and protected as a species of European importance in all the countries of the Eastern Baltic region (Gavrilova 2003, Kuusk et al. 2003, Sinkevičienė 2021, Suško et al. 2022, Sinkevičienė et al. 2023).

Over the last three decades, the research related to the implementation of the Directives of the European Union (Council Directive 92/43/EEC "Directive of Habitats" and Directive 2000/60/EC "Water Framework Directive") and protection of threatened species provided new data on the distribution of *Najas* species. Special attention was paid to the species of European importance, such as *Najas flexilis* (Sinkevičienė et al. 2023). The monitoring data revealed potential expansion of *N. marina* s.l. in different waterbodies, along with the need to implement protective measures for *N. minor* and *N. flexilis* and to more detailed studies of this specific genus.

The aim of our work was to present a mapped distribution of *Najas* species in the territory of Lithuania, based on the analysis of revised historical and recently collected herbarium specimens. For *Najas* specimens collected in the 19^th^ century, it was important to clarify the locations indicated on the handwritten labels as well as how precisely these locations were cited in contemporary and later literature sources. In the case of *Najas marina* L. s.l., it was important to assess the distribution of *Najas marina* L. and *Najas major* All. as separate species, thus comparing their expansion.

## Material and methods

In this paper, we follow the concept that *Najas marina* s.l. is represented by two species *Najas marina* and *N. major* as stated by Rüegg et al. (2019) and Kamaitytė-Bukelskienė et al. (In press). The species name *Najas flexilis* is used in a broad sense within the publication, as it is listed under this name in Annex II of the Habitats Directive. Collections in the herbaria of Lithuania (WI, BILAS) as well as recently collected specimens were examined. The morphological characteristics of the leaves were mainly used to identify the plants, as most of the specimens in the herbarium were sterile or had only unripe fruits. Taxonomic keys of Tzvelev (1979), Casper and Krausch (1980) and Troia (2022) were used for species identification. In some cases, leaf morphometric parameters of *Najas marina* s.l. were checked according to data by Rüegg et al. (2019).

Three study periods were distinguished for distribution mapping. The first period (1800–1850) covers the first half of the 19^th^ century. During this period, Vilnius Imperial University was open from 1803 to 1832, followed by the Academy of Medicine and Surgery from 1832 to 1842 after the University closure. The leading collectors and identifiers of aquatic plants were J. F. Wolfgang (1777–1859) and S. B. Gorski (1802–1868), who were both professors of pharmacy at the Vilnius Academy of Medicine and Surgery. After the closure of these institutions, botanical studies ceased, therefore no specimens are known from the second half of the 19^th^ century or the first half of the 20^th^ century.

During the latter period, the only known locality mentioned by Slawinski (1924) was Žalieji Ežerai Lake. Therefore, the second period includes data from the short time between 1950 and 1989. This period was characterised by increased attention to the investigation of aquatic vegetation of lakes (Snarskis and Adomavičiūtė 1954, Natkevičaitė 1954, Bagdonaitė 1960, Šarkinienė 1961, Bagdonaitė 1962, Galinis 1962, Šarkinienė 1963b, Šarkinienė 1968) and the genus *Potamogeton* (Galinis 1963). Main collectors of the herbarium specimens were professors and scientists (A. Bagdonaitė, V. Galinis, I. Šarkinienė, J. Balevičienė, Ž. Lazdauskaitė, V. Stepanavičienė (Tamošiūnaitė), and students V. Dabulskytė, J. V. Liubinavičius, V. Motiekaitytė).

The third period (1990–2025) represents the increased focus on endangered species and research related to implementing the Directives of the European Union (Habitats Directive and Water Framework Directive). The collectors of *Najas* species were A. Balevičius, A. Balsevičius, I. Bodnieks, R. Briškaitė, E. Bukelskis, J. Butkuvienė, Z. Gudžinskas, J. Karosienė, K. Katilius, V. Licite, E. Lakotko, V. Stukonis, Z. Sinkevičienė and L. Petrulaitis. All specimens were revised by L. Kamaitytė-Bukelskienė.

For the preparation of the distribution maps, the locations where the presence of the species is confirmed by herbarium specimens were used. If the literature data were confirmed by herbarium specimens collected during subsequent research, the sites were also mapped. All of the revised plant specimens of *Najas* are listed in Supplementary Table 1 (Suppl. material 1).

Maps were built using R software (R Core Team 2021) with packages *ggspatial* (Dunnington 2023) and *rnaturalearth* (Massicotte and South 2017).

## Results

### Najas marina L.

The first specimens of *Najas marina* were collected at the beginning of the 19^th^ century and stored in the herbarium of Vilnius University (WI). Only a few specimens are labelled with an indication of the place and time of collection, which is sometimes questionable.

**Figure 1.**
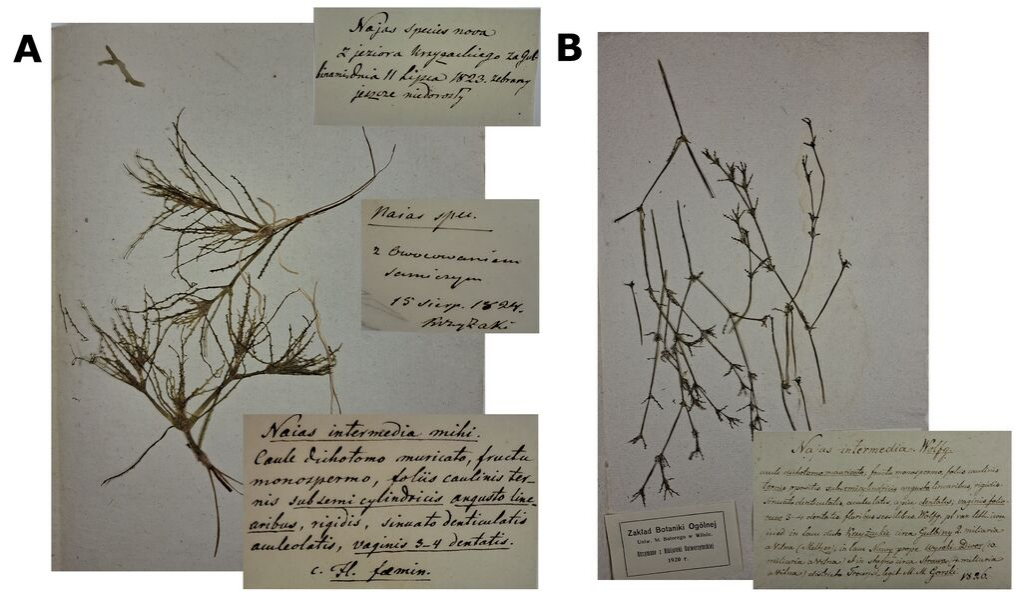
The specimens of *Najas marina* collected in the 19^th^ century: **A** the specimens collected in Kryžiokai Lake on 11 July 1823 and labelled as *Najas intermedia* mihi with original description by J. F. Wolfgang; **B** the specimens collected by S. B. Gorski in August 1823 in the Nava Lake and labelled as *Najas intermedia* Wolfg. The specimen selected as the lectotype of *Najas intermedia* Wolfg. ex Gorski by Brauchler (2015).

One herbarium sheet contained two specimens of plants at an early stage of development with three handwritten labels (Fig. 1A). One label with the inscription '*Najas* species nova' indicated the locality as "Kryžiokų Lake near Gulbinai" and the date of collection as "11 July 1823". The other label gives the plant name "*Naias intermedia* mihi" and a detailed morphological description. A third small label noted that the plants with fruit were found in the same lake on 15 August 1824. As the handwriting on the labels differs, they may have been written by more than one person, possibly at different times. One more sheet contained one plant specimen with a label bearing a morphological description and a note about the date of collection of the fruiting plants.

One of the specimens, judging by the handwritten label in Polish, was collected by Gorski in Nava Lake near Aukštadvaris on 03 August 1823 and named as *Najas monosperma* W. Two other labelled specimens, collected by Gorski at the same location and time, were named *Caulinia fragilis* Willd. Previously, they were found in the *N. minor* folder and re-identified as *N. marina* (Sinkevičienė 2001). One herbarium sheet shows tufted plants without developed fruits, while the other is presented by fruiting plants. Specimens that are morphologically very similar, however, without labels, are placed in approximately 30 unlabelled herbarium sheets. They were most likely collected for exchange, as similar specimens with a morphological description of "*Naias intermedia* mihi" and without any indication of locality were found stored in the National Herbarium of Ukraine (KW). A morphologically very similar herbarium specimen to that collected in Nava Lake was selected as the lectotype of *N. intermedia* Wolfg. ex Gorski (Bräuchler 2015) (Fig. 1B). The specimen labelled as *N. intermedia* Wolfg., with morphological description and indication of three localities: "Kryžiokų Lake near Gulbinai", Nava Lake near Wysoki-Dwor (Aukštadvaris) and "pond near Strėva". The label was possibly handwritten by Wolfgang in 1826. This date at the bottom of the label probably indicates when the label was written, but not when the plant was collected. We did not find any herbarium specimens collected in the above-mentioned vicinity of Strėva. The later publication by Gorski (1830), in which the species *N. intermedia* was validly published, did not refer to these three exact locations, but indicated "one lake near Vilnius and many other lakes in the Trakai district".

The publication by Wolfgang (1824) reported on a new species of *Najas* (with toothed leaf sheaths), which was collected by A. Jankiewicz in Lake Kryžiokai near Gulbinai. However, the discovery of a new *Najas* species in Nava Lake in 1823 was not included in the account of the rare and new species found that year, reported in the same publication.

Zelencow (1892) incorrectly reported the locality "pond near Strėva" as the locality of Najas major var. intermedia "in Strėva River". This erroneous information about locality was repeated by Snarskis (1954), Šarkinienė (1963a), Sinkevičienė (1992) and Sinkevičienė (2021). We did not find any herbarium specimens that could confirm the locations in Samanis Lake (Lazdijai District) reported by Šarkinienė (1961), Balevičius (1998) and lakes Tautesnis and Girininkija (Zarasai District) reported by Galinis (1958), Šarkinienė (1961) and Galinis (1962). Therefore, we cannot confirm which species, *N. marina* or *N. major*, was found in these lakes.

**Figure 2.**
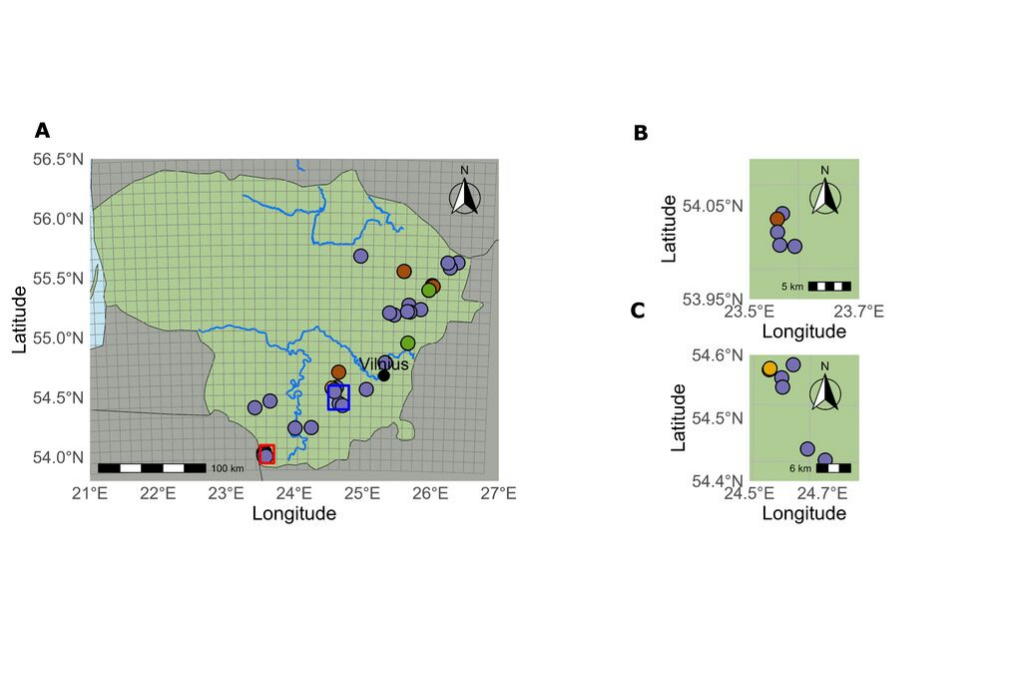
Distribution of *Najas marina* in Lithuania: **A** main map view; **B** within the red square in A; **C** within the blue square in A. Different colours represent different periods: dark green - the sites only recorded in the I period (until 1850); brown – the sites only recorded in the II period (1950–1989); purple – the sites only recorded in the III period (1990–2025); light green – the sites recorded in the II and III periods (1950–2025); yellow – the site, recorded in I (until 1850) and III periods (1990–2025).

During the revision of the specimens in both WI and BILAS Herbaria, 68 were identified as *Najas marina*. The occurrence of species was confirmed in 33 waterbodies. It was mostly found in lakes, but it has also been recorded in two reservoirs and one waterway connecting lakes. The distribution of this species is restricted to the lakes of the Baltic Uplands, which extend from the south to the northeast of the country (Fig. 2).

Herbarium data confirmed that *Najas marina* can persist in the same waterbody for centuries. This species has been present in lakes Kryžiokai (now known as Balsys Lake) and Nava (the recent Aukštadvaris Reservoir) since the early 19^th^ century. The first location was situated close to the City of Vilnius and, therefore, easily accessible. The other location was also relatively close to the capital, near former manor houses where the researchers could stay during their fieldwork.

Before 1990, *Najas marina* was found in nine lakes. Judging by the herbarium collections, only the populations in Lakes Baltelė and Bivainėlis (Ignalina District) were abundant. In general, the species was considered rare and Red-listed (Sinkevičienė 1992). As *N. marina* s.l. was protected, it is unclear to which species sensu stricto the literature records referred.

In the period after 1990, *Najas marina* locations have increased significantly. The species was only found repeatedly in the Lakes of Pravalas (Vilnius District) and Dringis (Ignalina District). The earlier site at Dringis Lake was known from literature (Šarkinienė 1961, Šarkinienė 1963b). In the advisory years, the species was found for the first time in many lakes (Dūkštas, Žaltytis, Žuvintas, Viešintas), which have been surveyed several times in the past or are included in the state monitoring programme. In lakes, such as Kauknorėlis, Rašia, Žaltytis and Žuvintas, populations were found particularly abundant, especially in the muddy, shallowest areas of these waterbodies. *N. marina* was found in different lakes from *N. major*. Only in one bay of Ilgis Lake (Varėna District) were both species found growing together.

### Najas major All.

**Figure 3.**
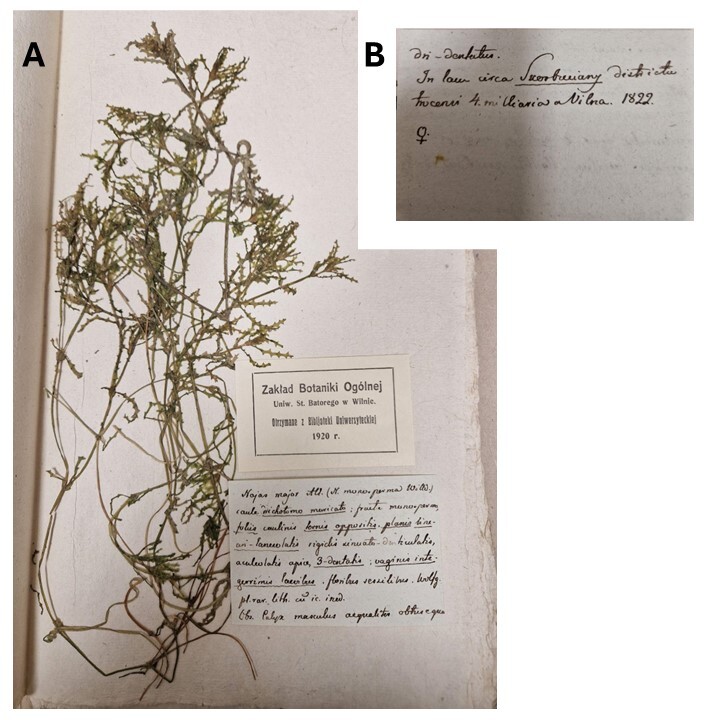
The first specimens of *Najas major*, collected in a lake near Skorbutėnai in 1822 and labelled by S. B. Gorski. **A** general view; **B** opposite side of the label.

The oldest specimens of *Najas major* collected in Lithuania at the beginning of the 19^th^ century were also found in the Herbarium of Vilnius University (WI). We found one specimen bearing a detailed label with characteristic Gorski's handwriting (Fig. 3). The species name *N. major* All. (*Najas monosperma* Willd.) and the morphological description were written on one side of the label. The indication of locality 'in lake circa Skorbuciany districtum Trocensi...' [in lake near Skorbutėnai in Trakai District...] and the collecting year 1822 were written on the opposite side.

Four more samples, which were morphologically similar and possibly collected in the same place, were unlabelled. One specimen was named as *Najas major* All. (*N. monosperma* Willd.), with a label that was probably handwritten by Wolfgang, containing the same morphological description and location, but was dated 1826. This date likely indicates when the label was written, rather than when the plants were collected. This finding was also reported in literature by Wolfgang (1822), that student A. Meltzer had found *N. monosperma* W. and *N. tetrasperma* W. near Skorbutėnai. We suspected that the unnamed lake near Skarbutėnai might be Ilgutis Lake near Lygainiai. However, *N. major* was not discovered until 2002, when *N. marina* was found there.

The second locality from the 19th century reported by Jundzilł (1830) was "lakes near Papiškės" (recent Šalčininkai District). Herbarium specimens collected 24.08.1824 are stored in the Herbarium of the (Polish) Academy of Sciences in Krakow (KRAM) (Köhler 1995).

Very few specimens of *Najas major* were collected during the period from 1950 to 1989.

**Figure 4.**
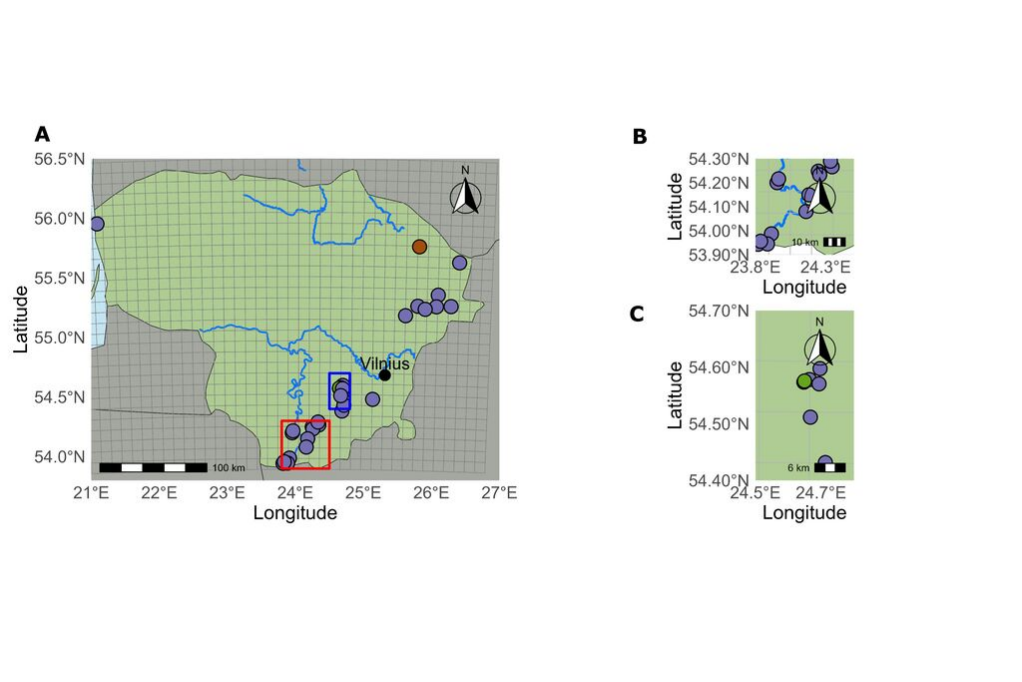
Distribution of *Najas major* in Lithuania: **A** main map view; **B** within the red square in A; **C** within the blue square in A. Different colours represent different periods: dark green – the site only recorded in the I period (until 1850); brown – the site only recorded in the II period (1950–1989); purple – the sites only recorded in the III period (1990–2025); light green – the sites recorded in the II and III periods (1950–2025).

In general, *Najas major* was documented in 22 waterbodies, according to the revised data (Fig. 4). Lakes are its main habitat, but it has also been recorded in a river, a reservoir and a sand quarry pond. Most sites are concentrated in the south and east of the country, with only one known site in the west. These sites are mainly located in the Lakelands of the Baltic Uplands, an area that extends from the southern part of the country to the north-east.

### Najas minor All.

**Figure 5.**
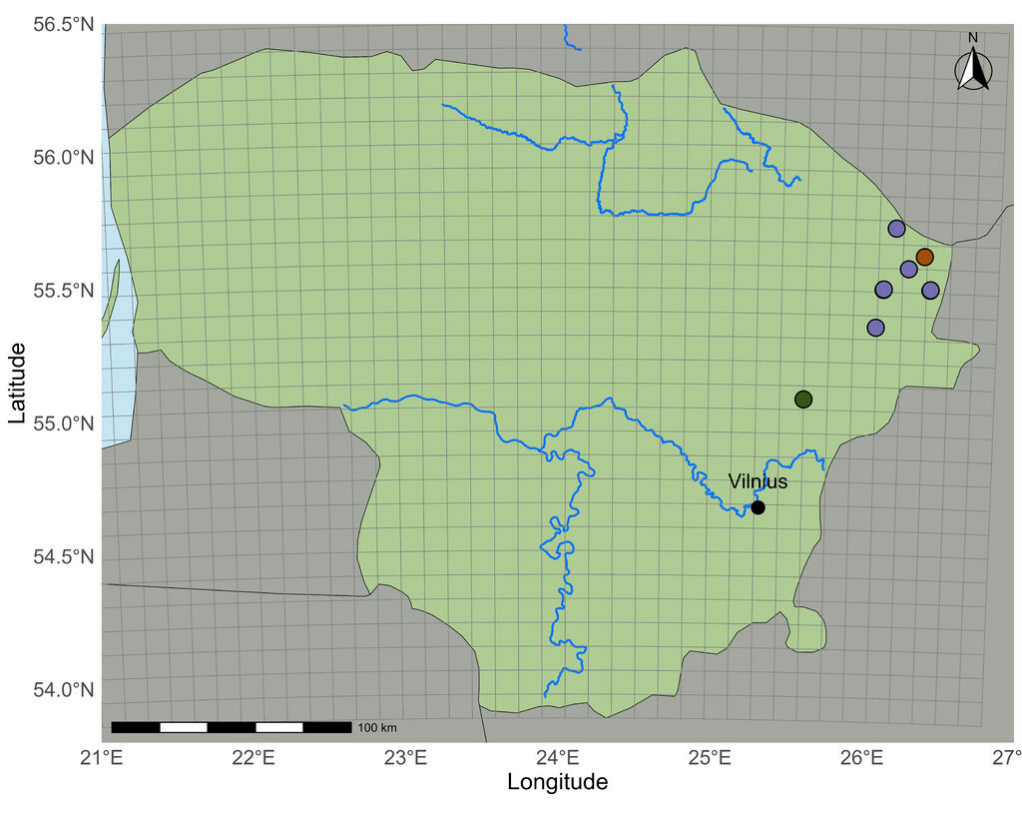
Distribution of *Najas minor* in Lithuania. Different colours represent different periods: dark green – the site only recorded in the I period (until 1850); brown – the site only recorded in the II period (1950–1989); purple – the sites only recorded in the III period (1990–2025).

The oldest labelled specimen of *Najas minor*, collected by S. B. Gorski in 1822, is preserved in the WI herbarium. Different 19^th^ century and later literature sources interpret the locations indicated on the specimen label differently. Information in the label is "In lacus Swinta circo Towiany, 8 miliaria a Vilna. Haciski et Meltzer primi inventores 1822" [In Lake Swinta, near Towiany, 8 miles from Vilna. Haciski and Meltzer, first inventors, 1822]. Wolfgang (1824) described the location in more detail. — …w iezierze pode dvorem Sędziego Towianskiego Szwinta zvanem" […in the lake called Švinta, near the manor of Judge Towianski]. Gorski (1830) re-interpreted information about locality as "sehr selten im See Godskischki, 8 Meilen von Wilna" [very rare in Lake Godskischki, 8 miles from Vilna]. At the end of the 19^th^ century, Zelencow (1892) incorrectly reported that Godskischki was located in Trakai County when it was in Vilnius County. This erroneous information was repeated by many researchers who failed to check the information on the herbarium labels. Later, the most important sources on the flora of Lithuania interpreted the name of the lake as Šventininkai Lake, located in the Trakai District (Snarskis 1954, Šarkinienė 1963a). This location was subsequently recorded in the Red Data Books (Sinkevičienė 1992, Sinkevičienė 2007). Although Sinkevičienė (2001) noted that the name of the Lake Szwinta (Šventas) cannot be interpreted as Šventininkai Lake, she incorrectly suggested that it could be Šventas Lake near Taučionys (Trakai District). The possible name of the Village Towiany, indicated on the specimen label, similar to "Towcany", made it difficult to identify the exact location, as it did not refer to a geographical name, but to the residence of the Towiany family (Anonymous 2025). The residence was in the settlement Antašventė. This fact helped establish the exact historical location of *N. minor*, which is Šventas Lake, situated in the Molėtai District (Fig. 5). The site mentioned by Gorski (1830) as "Godskischki" (recently Gačkiškiai) is located nearby.

During this research, the locality in Lake Šventas was not checked. The species was not found in Lake Skritelis, where it was recorded in 1961. Currently, *Najas minor* is known from five lakes: Alksnas, Dringis, Dūkštas, Sągardas (also known as Sangardas), Ažvintis and Zarasas. The location in Sągardas Lake has been known since 1998. The species continues to occur intermittently there to this day. In other lakes, *N. minor* has been recorded in recent decades. The species was first discovered in Lake Alksnas in 2006 and recent observations suggest that it is now spreading at the site. The species was first discovered in Lake Dūkštas in 2014 and is still present today. The species was recorded in Zarasas Lake twice, in 2015 and again in 2021. Although Dringis Lake was studied by several researchers in the 20^th^ century (Bagdonaitė 1962, Stepanavičienė 1991), *N. minor* was never recorded. The species was discovered in 2015 and remains abundant in the south-eastern part of the lake. The species has been listed on the Red List since 1992 and all known localities are supported by herbarium specimens (Sinkevičienė 1992, Sinkevičienė 2007, Rašomavičius 2021).

This study identified specimens of *Najas minor* in seven lakes. Although the oldest site in Šventas Lake is somewhat distant from the other localities, they are all concentrated within a relatively narrow area in the north-eastern part of the country, mainly in the Zarasai and Ignalina Districts.

### Najas flexilis (Willd.) Rostk. & Schmidt.

We did not find 19^th^-century specimens of *Najas flexilis* in any of the studied herbaria. However, botanical literature from the 20^th^ century (Dagys et al. 1934, Snarskis 1954) suggested that *N. flexilis* could be found growing in Lithuania. Triest (1988) recorded the locality of *N. flexilis* in Lithuania as "Lithuania, Lacus Switez, 1898, Dybowski s.n. (K, LY-Gand.)" [Switez Lake], which is actually located in Belarus.

The oldest labelled specimen was from Germantas Lake, collected in 1966. Several *Najas flexilis* plants were found within a sheet of macroalgae *Chara aspera* collected by I. Trainauskaitė (Sinkevičienė 2001). However, the species was never re-discovered in this lake.

Later, in 1998, the species was found at two different sites in Sągardas Lake. In one site, it was growing together with *Najas minor*. For a long time, findings of both species in Lake Sągardas were scarce. An abundant mixed population of both species was recorded in 2023.

**Figure 6.**
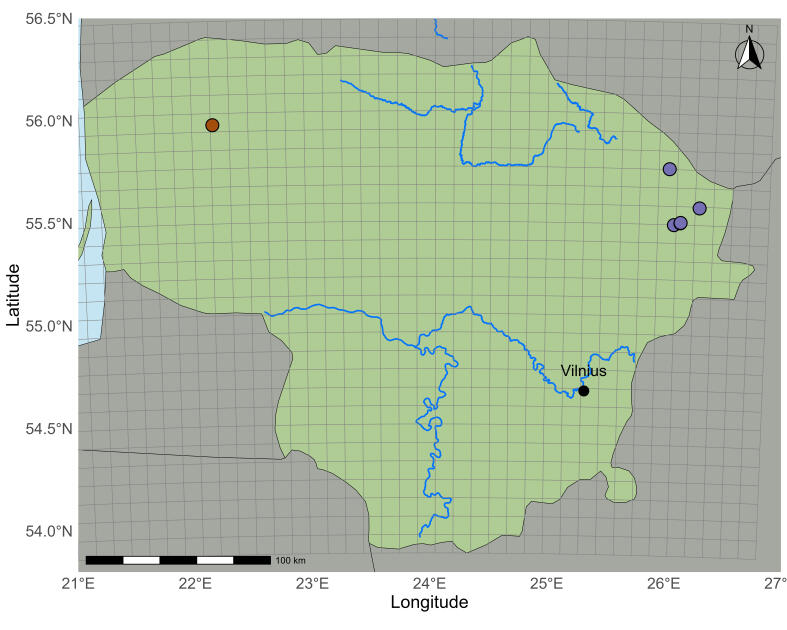
Distribution of *Najas flexilis* in Lithuania. Different colours represent different periods: brown– the site only recorded in the II period (1950–1989); purple – the sites only recorded in the III period (1990–2025).

Three new localities for the species were discovered during the 2020–21 period (Fig. 6). In 2020, the species was recorded at two sites in Dūkštas Lake. In 2021, it was found in Avilys Lake and a further locality was in Ažvintis Lake. This last lake is connected by a small channel to Sągardas Lake, where *Najas flexilis* was recorded previously, but the species was not found in Ažvintis Lake until 2021.

Except for a single, currently unconfirmed locality in the western part of the country, all the currently known records are concentrated in lakes in the north-eastern part of the country, which are relatively close together.

## Discussion

### Najas marina and Najas major

Both species have been known in Lithuania since the beginning of the 19^th^ century, but their distribution as separate species is poorly understood. Due to morphological similarities between species and their rarity, herbarium specimens collected between 1950 and 1990 were usually identified as *Najas marina* L. s.l., with no clear distinction between subordinate taxa (subspecies or varieties). Although two varieties (Snarskis 1954, Šarkinienė 1963a) or subspecies (Kuusk et al. 2003) have been mentioned in literature from this period, no clear morphological differences were indicated and the same locality was often recorded for both variations.

This study revealed a significant increase in the number of both *Najas* species locations over the last few decades. The diversity of waterbodies is also increasing. For instance, *N. major* was discovered in the Nemunas River and in a flooded sand quarry. However, both species are typically found in freshwater lakes, which are common in the southern and eastern parts of Lithuania. By contrast, in Latvia, *N. marina* is more common in brackish coastal lakes and the Gulf of Riga, but it is also found in freshwater lakes and gravel and dolomite quarries (Suško et al. 2022). *N. major* is widespread in natural freshwater lakes in south-eastern Latvia and it appears to be expanding its range northwards through the Baltic uplands.

The abundance and spread of *Najas marina* and *N. major* in bodies of water is not limited to Lithuania. The problems related to the spread of these species and their hybrids, as well as those regarding their delimitation, were studied in Germany (Rüegg et al. 2019).

The number of abundant populations of both species also increased during the last decades. Although the plants were not found in several waterbodies repeatedly, there are locations where they have survived for almost two centuries. Furthermore, both species, with one exception, were found only in separate waterbodies. Additionally, *Najas marina* was found more frequently, almost twice as often as the *N. major*. The distribution trends suggest that these species are not endangered and, therefore, do not require protection. On the contrary, their distribution and impact on aquatic plant communities should be monitored.

### Najas minor and Najas flexilis

Although the increase in the number of known localities of *N. minor* and *N. flexilis* in Lithuanian waters over the last two decades could suggest an improvement in the state of the populations, this should be treated with caution. We argue that the current state of the populations should be used as a baseline for future evaluations of the species. The increase in the number of known localities is primarily the result of greater attention being paid to searching for new localities and the special research of protected species, particularly those of European Union interest. The inclusion of aquatic macrophytes in national water monitoring programmes also had a positive impact on the better understanding of the distribution of Red-listed species.

Therefore, although *Najas minor* has been known since the beginning of the 19^th^ century, and *N. flexilis* was only discovered in the mid-20^th^ century, both species remain amongst the rarest in Lithuania and are Red-listed (Rašomavičius 2021). *N. flexilis* is endangered worldwide and is listed in the Annexes II and IV of the European Council Directive 92/43/EEC. The native range of *N. minor* is temperate and subtropical Eurasia to North Africa, while *N. flexilis* is native to the whole Nordic-temperate Northern Hemisphere (Casper 1979, POWO 2025). Therefore, the ranges of both species overlap in the Baltic States, but *N. minor* is not found in Estonia (Kuusk et al. 2003). The previous inventory of the species of European importance (Kamaitytė-Bukelskienė et al. 2022, Sinkevičienė et al. 2023) and this research confirmed the occurrence of *N. flexilis* in four localities in Lithuania. They are concentrated in the north-eastern part of the country and are closely connected with the main localities in Latvia. The number of lakes in Lithuania is negligible compared to the 19 extant localities of this species recorded in neighbouring Latvia (Suško et al. 2022). In terms of the number of *N. flexilis* sites, Latvia is one of the richest countries in the European Union together with Great Britain, Ireland, Norway and Switzerland (Wingfield et al. 2004, Wingfield et al. 2006, Mjelde et al. 2012, Suško et al. 2022). The southernmost location of *N. flexilis* in Europe was recorded in Austria (Pall 2011). This species is extinct in Poland, which is located to the south of Lithuania (Zalewska 1999). Currently, *N. minor* is known from five localities in Lithuania (Kamaitytė-Bukelskienė et al. 2022). This is considerably lower than the 15 known localities in Latvia (Suško et al. 2022). *N. flexilis* was first discovered in Latvia at the beginning of the 20^th^ century, whereas *N. minor* was found much later (Suško 1991, Suško et al. 2022). This pattern may indicate that recent climate changes are favourable for the northward spread of *N. minor*, a thermophilous species that is more prevalent in southern regions.

## Supplementary Material

4702FA6D-F487-54C8-8B61-AF51112AA310 10.3897/BDJ.14.e174747.suppl1 Supplementary material 1 Supplementary Table 1 Data type List Brief description The list of the revised Najas specimens from Lithuania held in the herbaria of the State Scientific Research Institute Nature Research Centre (BILAS) and Vilnius University (WI). File: oo_1480120.xlsx https://binary.pensoft.net/file/1480120 Kamaitytė-Bukelskienė, L., Sinkevičienė, Z.
